# Safety and effectiveness of post-trastuzumab deruxtecan regimens in patients with HER2-positive metastatic breast cancer who discontinued trastuzumab deruxtecan due to interstitial lung disease

**DOI:** 10.1016/j.breast.2026.104722

**Published:** 2026-02-04

**Authors:** Junji Tsurutani, Kazuki Nozawa, Toru Mukohara, Tetsuhiko Taira, Akiyo Yoshimura, Shigenori E. Nagai, Jun Hashimoto, Kazuo Matsuura, Toshiro Mizuno, Yoshiaki Shinden, Mitsugu Yamamoto, Toshimi Takano, Makoto Wakahara, Hirofumi Terakawa, Takashi Yamanaka, Yasuyuki Kojima, Takahiro Nakayama, Yuji Hirakawa, Kazuhito Shiosakai, Hiroji Iwata

**Affiliations:** a Advanced Cancer Translational Research Institute, Showa Medical University, 1-5-8 Hatanodai, Shinagawa-ku, Tokyo, 142-8555, Japan; b Department of Advanced Clinical Research and Development, Nagoya City University Graduate School of Medical Sciences, 1 Kawasumi, Mizuho-cho, Mizuho-ku, Nagoya, Aichi, 467-8601, Japan; c Department of Medical Oncology, National Cancer Center Hospital East, 6-5-1 Kashiwanoha, Kashiwa, Chiba, 277-8577, Japan; d Department of Oncology, Sagara Hospital, 3-31 Matsubaracho, Kagoshima, Kagoshima, 892-0833, Japan; e Department of Breast Oncology, Aichi Cancer Center, 1-1 Kanokoden, Chikusa-ku, Nagoya, Aichi, 454-8681, Japan; f Division of Breast Oncology, Saitama Cancer Center, 780 Komuro, Inamachi, Kita-Adachi-gun, Saitama, 362-0806, Japan; g Division of Medical Oncology, St. Luke's International Hospital, 9-1 Akashi-cho, Chuo-ku, Tokyo, 104-8560, Japan; h Department of Breast Oncology, Saitama Medical University International Medical Center, 1397-1 Yamane, Hidaka, Saitama, 350-1298, Japan; i Department of Medical Oncology, Mie University Hospital, 2-174 Edobashi, Tsu, Mie, 514-8507, Japan; j Department of Breast and Thyroid Surgery, Kagoshima University Graduate School of Medical and Dental Sciences, 8-35-1 Sakuragaoka, Kagoshima, Kagoshima, 890-8544, Japan; k Department of Breast Oncology, Hokkaido Cancer Center, 2-3-54 Kikusui shi-jo, Shiroishi-ku, Sapporo, Hokkaido, 003-0804, Japan; l Department of Breast Medical Oncology, Cancer Institute Hospital of Japanese Foundation for Cancer Research, 3-8-31 Ariake, Koto-ku, Tokyo, 135-8550, Japan; m Department of Breast and Endocrine Surgery, Tottori University Hospital, 36-1 Nishi-cho, Yonago, Tottori, 683-8504, Japan; n Department of Breast Surgery, Kanazawa University Hospital, 13-1 Takaramachi, Kanazawa, Ishikawa, 920-8641, Japan; o Department of Breast Surgery and Oncology, Kanagawa Cancer Center, 2-3-2 Nakao, Asahi-ku, Yokohama, Kanagawa, 241-8515, Japan; p Institute for Clinical Genetics and Genomics, Showa Medical University, 1-5-8 Hatanodai, Shinagawa-ku, Tokyo, 142-8555, Japan; q Department of Breast and Endocrine Surgery, Osaka International Cancer Institute, 3-1-69 Otemae, Chuo-ku, Osaka, Osaka, 541-8567, Japan; r Oncology Medical Science Department I, Daiichi Sankyo Co., Ltd., 3-5-1 Nihonbashi-honcho, Chuo-ku, Tokyo, 103-8426, Japan; s Data Intelligence Department, Daiichi Sankyo Co., Ltd., 1-2-58 Hiromachi, Shinagawa-ku, Tokyo, 140-8710, Japan

**Keywords:** Interstitial lung disease, Metastatic breast cancer, Trastuzumab deruxtecan, Subsequent treatment

## Abstract

**Background:**

The real-world EN-SEMBLE (jRCT1030220506) study investigated the effectiveness and safety of treatments subsequent to trastuzumab deruxtecan (T-DXd) in patients with human epidermal growth factor receptor 2 (HER2)-positive metastatic breast cancer (mBC). This post hoc analysis provides informative data for subsequent treatment in patients who discontinue T-DXd due to interstitial lung disease (ILD).

**Methods:**

Patients in EN-SEMBLE who discontinued T-DXd due to ILD were included in this analysis. Outcomes for first post-T-DXd treatment were real-world progression-free survival (rwPFS), real-world time to treatment failure (rwTTF), real-world time to next treatment (rwTTNT), and overall survival. ILD recurrence/exacerbation after initiating the first and second post-T-DXd treatments was evaluated.

**Results:**

This analysis included 146 patients. ILD grade during prior T-DXd treatment was 1, 2, 3, and unknown for 78 (53.4%), 46 (31.5%), 18 (12.3%), and 4 patients (2.7%), respectively. Most patients (81.5%) received subsequent anti-HER2 treatment. rwPFS was 7.2 months (95% confidence interval, 5.4, 10.2), rwTTF 6.5 months (5.1, 9.1), rwTTNT 7.8 months (5.9, 10.8), and overall survival 32.4 months (21.3, not estimable). These outcomes were numerically longer in patients with grade 1 versus 3 ILD during prior T-DXd treatment. Five patients (3.4%) had ILD recurrence during the first post-T-DXd treatment. Among the 93 patients who received second post-T-DXd treatment, 2 (2.2%) experienced ILD recurrence.

**Conclusion:**

These findings highlight the importance of early ILD detection and management so patients can receive, and potentially benefit from, subsequent anti-HER2 targeted therapies. ILD recurrence/exacerbation during subsequent therapies was low in patients who experienced ILD with T-DXd.

## Highlights

• 81.5% of patients received anti-HER2 treatment after discontinuing T-DXd due to ILD.
• Effectiveness outcomes were longest in those with grade 1 ILD with prior T-DXd.
• During the first post-T-DXd treatment, 5/146 patients (3.4%) had ILD recurrence.
• This analysis highlights the importance of early ILD detection with T-DXd in mBC.

## 1 Introduction

Trastuzumab deruxtecan (T-DXd) was first approved as third-line or later-line treatment for human epidermal growth factor receptor 2 (HER2)-positive metastatic breast cancer (mBC) based on the results of the DESTINY-Breast01 study [1], then for second-line treatment based on DESTINY-Breast03 [2]. T-DXd is currently recommended as standard of care in the second-line setting in Japanese, European, and American treatment guidelines [[3], [4], [5]].

In the initial report of the phase 2 DESTINY-Breast01 trial, the incidence of interstitial lung disease (ILD) was 13.6% [1], and subsequent clinical trials of mBC have reported an incidence ranging from 11.4% to 16.7% [[6], [7], [8], [9], [10]]. A pooled analysis of nine T-DXd trials across multiple tumor types found an incidence rate of 15.4% for ILD/pneumonitis [11]. Although most cases were mild (grade 1 or 2), 2.2% of patients with ILD/pneumonitis died [11]. ILD has emerged as an adverse reaction associated with T-DXd. Additionally, although the underlying mechanism is not understood, several reports found a higher risk of T-DXd-related ILD/pneumonitis among Japanese patients compared with patients from other countries [11,12].

Drug-induced ILD is a serious adverse event that can lead to fatal outcomes. As such, careful monitoring, prompt diagnosis, and immediate treatment are essential for ILD management [13,14]. Permanent discontinuation of T-DXd is recommended for patients with grade ≥2 ILD. At the time of T-DXd approval in Japan, permanent cessation of T-DXd was also mandated for patients presenting with grade ≥1 ILD/pneumonitis. Currently, it is recommended that patients with grade 1 ILD discontinue T-DXd treatment, which can be restarted at a reduced dose level under certain circumstances [13,15]. Drug-induced ILD is not unique to T-DXd or breast cancer treatment, as other anti-cancer agents are also associated with an increased risk of ILD [[16], [17], [18], [19]]. Considering this, it is important to understand whether subsequent anti-cancer treatments can be safely administered to patients who have experienced drug-induced ILD on T-DXd. Data assessing treatment outcomes with subsequent therapies following discontinuation of T-DXd treatment due to ILD in Japanese patients at high risk for ILD can serve as a benchmark for management of patients who develop ILD on T-DXd treatment in other countries.

The determination of appropriate treatment sequences for patients who discontinue T-DXd due to ILD is thus an important clinical question. The EN-SEMBLE study (jRCT1030220506) was designed to address the gap in data regarding the appropriate treatment sequence for patients with mBC following discontinuation of T-DXd due to progressive disease (PD), adverse events, or ILD [20]. This subgroup analysis of patients in the EN-SEMBLE study who discontinued T-DXd due to ILD was conducted to provide detailed data to help manage subsequent treatment in these patients.

## 2 Patients and methods

### 2.1 Study design and patients

EN-SEMBLE was an observational, real-world, nationwide study conducted at 222 sites in Japan to investigate subsequent treatments after discontinuation of T-DXd therapy for any reason in patients with HER2-positive mBC. Most patients (92.3%) in the study received T-DXd as third-line or later-line treatment after developing resistance to trastuzumab emtansine [20]. The full EN-SEMBLE study methodology, including patient eligibility criteria, has been published [20]. Patients who met the eligibility criteria between May 25, 2020 and May 31, 2023 were enrolled in the EN-SEMBLE study and followed from the initiation of subsequent treatment after discontinuing T-DXd until November 30, 2023 [20]. This post hoc analysis included patients who were enrolled in the EN-SEMBLE study and discontinued T-DXd treatment due to ILD. ILD that occurred during T-DXd treatment was graded by the attending physician according to the Common Terminology Criteria for Adverse Events v5.0. ILD monitoring and management were performed at the discretion of the attending physicians during routine clinical practice, based on guidelines and the package insert [15,[21], [22], [23]].

The EN-SEMBLE study was conducted in accordance with the principles outlined in the Declaration of Helsinki (revised 2013) and the relevant Japanese ordinance. The study was approved by the Showa University Research Ethics Review Board on October 26, 2022 (reference number: 21000128). All participants provided written informed consent or were allowed to opt out.

### 2.2 Outcomes

The following outcomes were assessed for the first post-T-DXd treatment: real-world progression-free survival (rwPFS), real-world time to treatment failure (rwTTF), real-world time to next treatment (rwTTNT), and overall survival (OS). Detailed descriptions of these outcome measures were reported previously [20]. Recurrence/exacerbation of ILD after initiating the first and second post-T-DXd treatment is also reported.

### 2.3 Statistical analysis

No sample size was calculated for this post hoc analysis. The data cutoff date was November 30, 2023. The data were analyzed for the total population, by ILD grade, and by first post-T-DXd treatment regimen. Descriptive statistics were reported as follows: categorical variables were summarized using frequency and proportion, and continuous variables were summarized using medians with quartiles. Kaplan–Meier curves were generated for the post-T-DXd outcomes; median and 95% confidence intervals (CIs) were calculated using the Kaplan–Meier and the Brookmeyer–Crowley methods, respectively. Hypothesis testing was conducted with a two-sided significance level of 5% and 95% CIs were calculated for interval estimations. There were no adjustments for multiplicity and missing data were not imputed. SAS version 9.4 (SAS Institute; Cary, NC, USA) was used for all statistical analyses.

## 3 Results

### 3.1 Patients

**Fig. 1:**
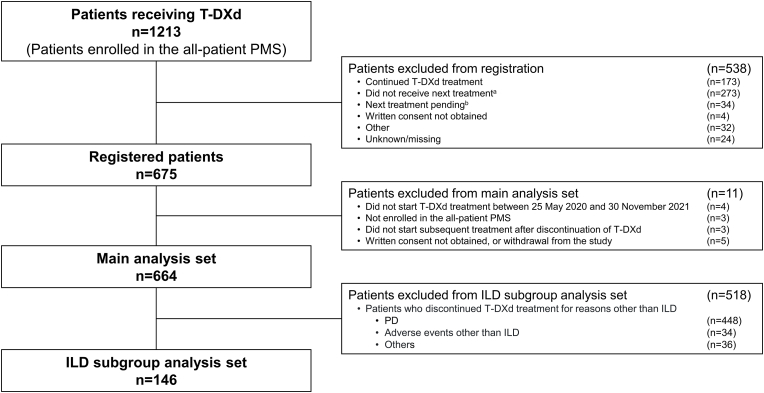
Patient disposition ^a^Death, palliative care, lost to follow-up, or transferred to another hospital after discontinuation of T-DXd. ^b^Did not start next treatment after discontinuation of T-DXd. ILD, interstitial lung disease; PD, progressive disease; PMS, post-marketing surveillance; T-DXd, trastuzumab deruxtecan.

Of the 664 patients included in the EN-SEMBLE study, 146 patients (22.0%) discontinued T-DXd due to ILD and were included in the current analysis (Fig. 1). Patient characteristics are shown in Table 1. Of the 146 included patients (ILD subgroup), 78 (53.4%) had grade 1 ILD, 46 (31.5%) had grade 2 ILD, 18 (12.3%) had grade 3 ILD, and no patients had grade 4 or 5 ILD (Table 1). There were four patients for whom the ILD grade was unknown. The median (Q1, Q3) age was 63 (55, 70) years, 115 patients (78.8%) had visceral metastasis, and the median (Q1, Q3) number of treatment lines prior to T-DXd was 3.5 (2, 5). There were no major differences in these factors at the start of the first post-T-DXd treatment across ILD grades.

**Table 1:** Patient characteristics.

	ILD subgroup (any grade)	ILD grade for discontinuation of T-DXd treatment^a^^,^^b^		
		Grade 1	Grade 2	Grade 3
No. patients, n (%)	146 (100)	78 (53.4)	46 (31.5)	18 (12.3)

**At the start of the first post-T-DXd treatment**				

Age, years, median (Q1, Q3)	63 (55, 70)	62.5 (55, 70)	64.5 (56, 69)	61.5 (51, 71)
Sex				
Female	144 (98.6)	77 (98.7)	46 (100)	17 (94.4)
Male	2 (1.4)	1 (1.3)	0	1 (5.6)
HER2 status				
IHC3+	103 (70.5)	59 (75.6)	30 (65.2)	11 (61.1)
IHC2+/ISH+	35 (24.0)	16 (20.5)	15 (32.6)	4 (22.2)
Other ISH+/ISH-	3 (2.1)	2 (2.6)	0	1 (5.6)
Unknown	5 (3.4)	1 (1.3)	1 (2.2)	2 (11.1)
ECOG-PS				
0	74 (50.7)	41 (52.6)	25 (54.3)	7 (38.9)
1	49 (33.6)	26 (33.3)	15 (32.6)	8 (44.4)
≥2	15 (10.3)	7 (9.0)	6 (13.0)	2 (11.1)
Unknown	8 (5.5)	4 (5.1)	0	1 (5.6)
Hormone-receptor				
Positive	95 (65.1)	45 (57.7)	34 (73.9)	13 (72.2)
Negative	49 (33.6)	32 (41.0)	12 (26.1)	4 (22.2)
Unknown	2 (1.4)	1 (1.3)	0	1 (5.6)
Visceral metastasis				
Present	115 (78.8)	57 (73.1)	40 (87.0)	15 (83.3)
Absent	31 (21.2)	21 (26.9)	6 (13.0)	3 (16.7)
Brain metastasis				
Present	38 (26.0)	20 (25.6)	13 (28.3)	4 (22.2)
Absent	103 (70.5)	55 (70.5)	33 (71.7)	12 (66.7)
Unknown	5 (3.4)	3 (3.8)	0	2 (11.1)
No. lines of therapy prior to T-DXd initiation, median (Q1, Q3)	3.5 (2, 5)	4 (3, 5)	3 (2, 5)	3 (2, 5)

**T-DXd treatment details**				

mBC status (at T-DXd initiation)				
De novo stage IV	50 (34.2)	31 (39.7)	12 (26.1)	5 (27.8)
Recurrent breast cancer	91 (62.3)	44 (56.4)	33 (71.7)	12 (66.7)
Others	3 (2.1)	3 (3.8)	0	0
Unknown	2 (1.4)	0	1 (2.2)	1 (5.6)
Duration of T-DXd treatment, months, median (Q1, Q3)	6.2 (3.7, 9.7)	6.4 (3.7, 9.9)	6.2 (4.2, 9.9)	4.2 (2.8, 8.4)
Best overall response to T-DXd treatment				
Objective response rate	98 (67.1)	58 (74.4)	28 (60.9)	9 (50.0)
Complete response	9 (6.2)	6 (7.7)	3 (6.5)	0
Partial response	89 (61.0)	52 (66.7)	25 (54.3)	9 (50.0)
Stable disease	42 (28.8)	18 (23.1)	15 (32.6)	8 (44.4)
Progressive disease	2 (1.4)	0	1 (2.2)	1 (5.6)
Not evaluable	4 (2.7)	2 (2.6)	2 (4.3)	0
Time from T-DXd discontinuation date to the first date of the first post-treatment, days, median (Q1, Q3)	50.5 (23, 90)	43 (21, 67)	66 (35, 114)	60 (50, 153)
Steroid administration for ILD induced by T-DXd				
Yes	72 (49.3)	14 (17.9)	38 (82.6)	17 (94.4)
None	74 (50.7)	64 (82.1)	8 (17.4)	1 (5.6)
Resolution of ILD at the first date of the first post-T-DXd treatment				
Resolved	101 (69.2)	56 (71.8)	30 (65.2)	12 (66.7)
Not resolved	42 (28.8)	22 (28.2)	14 (30.4)	5 (27.8)
Unknown	3 (2.1)	0	2 (4.3)	1 (5.6)

Data are shown as n (%) unless otherwise noted.

ECOG-PS, Eastern Cooperative Oncology Group performance score; HER2, human epidermal growth factor receptor 2; IHC, immunohistochemistry; ILD, interstitial lung disease; ISH, in situ hybridization; mBC, metastatic breast cancer; Q, quartile; T-DXd, trastuzumab deruxtecan.

a Grade determined by the attending physician.

b There were no patients with known grade 4 or 5 ILD; the ILD grade of four patients was unknown.

T-DXd treatment duration was similar for patients with grade 1 and grade 2 ILD (6.4 months and 6.2 months, respectively), and shorter for those with grade 3 ILD (4.2 months). The objective response rate to T-DXd treatment was greatest for patients with grade 1 ILD (74.4% [58/78]), followed by grade 2 ILD (60.9% [28/46]) and grade 3 ILD (50.0% [9/18]). The proportion of patients who received steroids was lowest for those with grade 1 ILD (17.9% [14/78]) and notably higher for patients with grade 2 ILD (82.6% [38/46]) and grade 3 ILD (94.4% [17/18]). The proportion of patients whose ILD had resolved at the initiation of the first post-T-DXd treatment was highest for those with grade 1 ILD (71.8% [56/78]); the proportion was similar among patients with grade 2 and grade 3 ILD (65.2% [30/46] and 66.7% [12/18], respectively).

### 3.2 First post-T-DXd treatment

The most common first post-T-DXd treatment was anti-HER2-containing regimens (81.5% [119/146]). Endocrine therapy ± CDK4/6 inhibitors (CDK4/6i) and chemotherapy were administered in 11.0% [16/146] and 6.8% [10/146] of patients, respectively (Supplementary Table 1). These trends were similar among patients with grade 1, 2, and 3 ILD.

### 3.3 Post-T-DXd outcomes

**Fig. 2:**
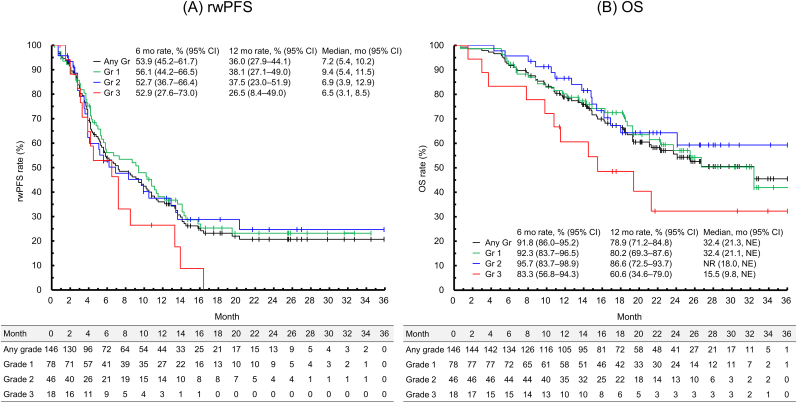
Kaplan–Meier curves of rwPFS (A) and OS (B) by ILD grade (time from the date of the first post-T-DXd treatment) CI, confidence interval; Gr, grade; ILD, interstitial lung disease; mo, month; OS, overall survival; PFS, progression-free survival; rwPFS, real-world PFS; T-DXd, trastuzumab deruxtecan.

The median (Q1, Q3) follow-up was 17.5 months (11.4, 24.8) for the entire ILD subgroup, and 18.5 months (11.8, 25.5), 16.7 months (12.4, 24.2), and 15.0 months (9.8, 21.3) for patients with grade 1, 2, and 3 ILD, respectively (Table 2). For the entire ILD subgroup, the rwPFS was 7.2 months (95% CI, 5.4, 10.2), rwTTF was 6.5 months (95% CI, 5.1, 9.1), rwTTNT was 7.8 months (95% CI, 5.9, 10.8), and OS was 32.4 months (95% CI, 21.3, not estimable [NE]) (Fig. 2 and Table 2). The rwPFS, rwTTF, and rwTTNT were best for patients with grade 1 ILD, followed by grade 2 and 3 ILD. OS for patients with grade 3 ILD was shorter than for patients with grade 1 or 2 ILD.

**Table 2:** Outcomes for first post-T-DXd treatment by ILD grade.

	ILD subgroup (any grade)	ILD grade for discontinuation of T-DXd treatment^a^^,^^b^		
		Grade 1	Grade 2	Grade 3
No. patients, n (%)	146 (100)	78 (53.4)	46 (31.5)	18 (12.3)

Follow-up period, months median (Q1, Q3)	17.5 (11.4, 24.8)	18.5 (11.8, 25.5)	16.7 (12.4, 24.2)	15.0 (9.8, 21.3)

rwPFS	7.2 (5.4, 10.2)	9.4 (5.4, 11.5)	6.9 (3.9, 12.9)	6.5 (3.1, 8.5)
rwTTF	6.5 (5.1, 9.1)	7.3 (5.3, 11.3)	6.1 (3.8, 10.9)	5.7 (3.1, 7.2)
rwTTNT	7.8 (5.9, 10.8)	10.7 (5.8, 14.3)	7.6 (4.9, 13.4)	7.1 (3.8, 11.3)
OS	32.4 (21.3, NE)	32.4 (21.1, NE)	NR (18.0, NE)	15.5 (9.8, NE)

Data are shown as median (95% CI) unless otherwise noted.

Median and 95% CIs were calculated using the Kaplan–Meier and Brookmeyer–Crowley methods, respectively.

CI, confidence interval; ILD, interstitial lung disease; NE, not estimable; NR, not reached; OS, overall survival; Q, quartile; rwPFS, real-world progression-free survival; rwTTF, real-world time to treatment failure; rwTTNT, real-world time to next treatment; T-DXd, trastuzumab deruxtecan.

a Grade determined by the attending physician.

b There were no patients with known grade 4 or 5 ILD; the ILD grade of four patients was unknown.

**Fig. 3:**
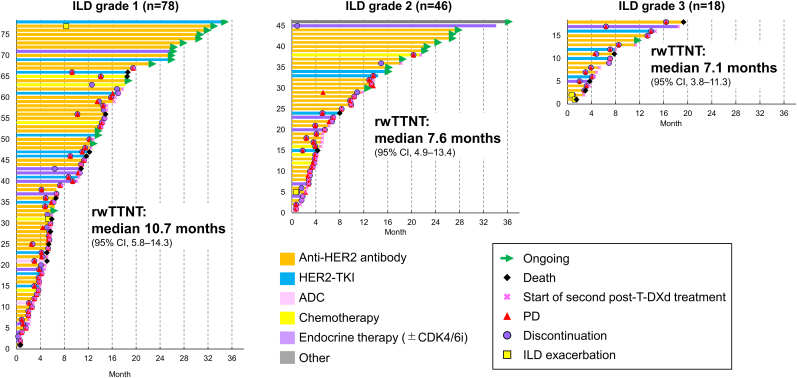
Swimmer plot of rwTTNT by ILD grade and first post-T-DXd treatment ADC, antibody–drug conjugate; CI, confidence interval; HER2, human epidermal growth factor receptor 2; ILD, interstitial lung disease; PD, progressive disease; rwTTNT, real-world time to next treatment; T-DXd, trastuzumab deruxtecan; TKI, tyrosine kinase inhibitor.

The rwTTNT by ILD grade and first post-T-DXd treatment for each patient is shown in Fig. 3 and Table 2. For patients with grade 1, 2, and 3 ILD, the median rwTTNT was 10.7 months (95% CI, 5.8, 14.3), 7.6 months (95% CI, 4.9, 13.4), and 7.1 months (95% CI, 3.8, 11.3), respectively.

By first post-T-DXd treatment regimen, patients treated with anti-HER2 antibody therapy had a rwPFS of 8.5 months (95% CI, 5.3, 12.2), rwTTF of 6.9 months (5.0, 11.0), rwTTNT of 8.3 months (5.8, 12.9), and OS of 26.7 months (19.3, NE) (Table 3). Patients treated with a HER2-tyrosine kinase inhibitor had a rwPFS of 8.9 months (4.3, 13.6), rwTTF of 8.9 months (4.3, 13.6), and rwTTNT of 11.1 months (5.2, 17.3); OS was not reached (11.7, NE) (Table 3).

**Table 3:** Outcomes for first post-T-DXd treatment by subsequent treatment regimen.

	ILD subgroup	Anti-HER2 antibody	HER2-TKI	ADC	Chemotherapy	Endocrine therapy^a^
No. patients, n (%)	146 (100)	96 (65.8)	20 (13.7)	3 (2.1)	10 (6.8)	16 (11.0)

rwPFS	7.2 (5.4, 10.2)	8.5 (5.3, 12.2)	8.9 (4.3, 13.6)	2.1 (0.7, NE)	4.9 (0.7, 14.3)	6.5 (2.0, 9.4)
rwTTF	6.5 (5.1, 9.1)	6.9 (5.0, 11.0)	8.9 (4.3, 13.6)	2.1 (0.7, NE)	4.6 (0.7, 14.1)	4.7 (1.2, 6.7)
rwTTNT	7.8 (5.9, 10.8)	8.3 (5.8, 12.9)	11.1 (5.2, 17.3)	2.8 (1.0, NE)	5.4 (0.7, 18.5)	6.1 (2.1, 10.7)
OS	32.4 (21.3, NE)	26.7 (19.3, NE)	NR (11.7, NE)	10.9 (5.1, NE)	18.5 (0.7, NE)	NR (19.3, NE)

Data are shown as median (95% CI) unless otherwise noted.

One patient received treatment that was classified into the other regimen category; however, medians (95% CIs) for rwPFS, rwTTF, rwTTNT, and OS were not estimated.

ADC, antibody–drug conjugate; CI, confidence interval; HER2, human epidermal growth factor receptor 2; ILD, interstitial lung disease; NE, not estimable; NR, not reached; OS, overall survival; rwPFS, real-world progression-free survival; rwTTF, real-world time to treatment failure; rwTTNT, real-world time to next treatment; T-DXd, trastuzumab deruxtecan; TKI, tyrosine kinase inhibitor.

a Monotherapy or with CDK4/6i.

### 3.4 Recurrence/exacerbation of ILD

During the first post-T-DXd treatment period, 5/146 patients (3.4%) experienced ILD recurrence/exacerbation (Table 4). By ILD grade at the time of T-DXd treatment, 2/78 patients (2.6%) with grade 1 ILD, 1/46 (2.2%) with grade 2 ILD, and 2/18 (11.1%) with grade 3 ILD had recurrence/exacerbation of ILD with their first post-T-DXd treatment. The times from the initiation of the next treatment to ILD recurrence/exacerbation for each of these five patients were 22 days (grade 2 ILD), 23 days (grade 3), 39 days (grade 3), 161 days (grade 1), and 255 days (grade 1). By ILD status on day 1 of the first post-T-DXd treatment, ILD recurrence/exacerbation occurred in 3/101 patients with resolved status and 2/42 patients with not resolved status.

**Table 4:** Details of patients experiencing ILD recurrence/exacerbation during post-treatment period.

No.^a^	Age, years	mBC status	T-DXd treatment period					First post-T-DXd treatment period					Second post-T-DXd treatment period				
			T-DXd treatment		ILD information			First post-T-DXd	ILD incidence		RFD	BOR	Second post-T-DXd		ILD incidence		BOR
			Period, months	BOR	CTCAE grade	Steroid use	ILD status^b^	Regimen	Yes/No	Onset day			Yes/No	Regimen	Yes/No	Onset day	
**During the first post-T-DXd treatment period**																	

1	59	Recurrent	3.7	PR	3	Yes	Res	T + P + ERI	Yes	23	ILD	SD	Yes	Lapa + Cape	No	–	PD
2	60	*De novo*	7.9	SD	1	No	NRes	T	Yes	255	Ongoing	SD	No	–	–	–	–
3	73	Recurrent	5.6	PR	1	Yes	Res	ERI	Yes	161	ILD	SD	No	–	–	–	–
4	68	Recurrent	5.6	PR	2	Yes	NRes	T + ERI	Yes	22	ILD	SD	Yes	V	No	–	SD
5	81	Recurrent	14.1	SD	3	Yes	Res	T + P + ERI	Yes	39	ILD	Not evaluable	No	–	–	–	–

**During the second post-T-DXd treatment period**																	

1	66	*De novo*	19.5	PR	2	No	NRes	T	No	–	Others	SD	Yes	Lapa + Cape	Yes	43	SD
2	52	*De novo*	1.7	PR	1	No	NRes	T + V	No	–	PD	PD	Yes	T + Pacli	Yes	78	PD

BOR, best overall response; Cape, capecitabine; CTCAE, Common Terminology Criteria for Adverse Events; ERI, eribulin; ILD, interstitial lung disease; Lapa, lapatinib; mBC, metastatic breast cancer; NRes, not resolved; P, pertuzumab; Pacli, paclitaxel; PD, progressive disease; PR, partial response; Res, resolved; RFD, reason for discontinuation; SD, stable disease; T, trastuzumab; T-DXd, trastuzumab deruxtecan; V, vinorelbine.

a All patients were female.

b ILD status on day 1 of the first post-T-DXd treatment.

Among the 146 patients, 93 received a second post-T-DXd treatment. During the second post-T-DXd treatment period, 2/93 patients (2.2%) experienced ILD recurrence (Table 4). There was no overlap between the cases of ILD recurrence during the first and second post-T-DXd treatment periods. During the second post-T-DXd treatment period, four patients were re-administered T-DXd, and no ILD recurrence was observed in these patients.

## 4 Discussion

This subgroup analysis of the EN-SEMBLE study reported effectiveness outcomes and ILD recurrence/exacerbation with post-T-DXd treatment for 146 patients who received T-DXd treatment for mBC and experienced ILD in the real-world setting. Over 80% of patients received anti-HER2 therapy following T-DXd treatment. rwPFS, rwTTF, and OS with first post-T-DXd treatment were shortest in patients with grade 3 ILD and longest in patients with grade 1 ILD. Among the 146 patients included in the ILD subgroup, five had recurrent ILD with their first post-T-DXd treatment.

Among patients who discontinued T-DXd due to ILD, most received anti-HER2 therapy (81.5%) as their first post-T-DXd treatment. Although statistical significance was not evaluated, the distribution of first post-T-DXd treatment regimens was generally numerically similar across ILD grades. For the main analysis of the EN-SEMBLE study, patients who discontinued T-DXd due to PD received anti-HER2 therapy (69.6%), chemotherapy (11.2%), chemotherapy + bevacizumab (10.9%), or endocrine therapy ± CDK4/6i (5.8%) [20], indicating differences in the distribution of first post-T-DXd treatment according to the reason for T-DXd treatment discontinuation. Those who discontinued T-DXd due to ILD are considered to have switched to the next therapy before disease progression, which likely explains the higher proportion of patients continuing anti-HER2 therapy. Patients treated with an anti-HER2 antibody or a HER2 tyrosine kinase inhibitor showed numerically better outcomes compared with those on other regimens.

In the main analysis of the EN-SEMBLE study, effectiveness outcomes for heavily pre-treated patients who discontinued T-DXd due to PD were 3.5 months for rwPFS, 4.2 months for rwTTNT, and 12.0 months for OS [20]. Furthermore, patients who discontinued due to adverse events (including ILD) tended to have longer outcomes than those who discontinued due to PD, with no significant difference in the hazard ratios between ILD and non-ILD adverse events [20]. In the present subanalysis, rwPFS, rwTTNT, and OS were longer in patients who discontinued T-DXd due to ILD (7.2 months, 7.8 months, and 32.4 months, respectively) compared with those who discontinued T-DXd due to PD. Effectiveness outcomes were longest in those with grade 1 ILD with prior T-DXd therapy. There was a difference between patients with grade 1 and grade 3 ILD in the interval from discontinuation of T-DXd to initiation of the first post-T-DXd treatment. The prolonged recovery period required for grade 3 ILD may result in treatment delays that contribute to shorter rwPFS due to more pronounced disease relapse.

Although the number of cases was limited, the recurrence/exacerbation rate of ILD following T-DXd treatment appeared numerically higher in patients with grade 3 ILD, with 2 of 18 patients (11.1%) experiencing recurrence, compared to 2 of 78 (2.6%) with grade 1 ILD and 1 of 46 (2.2%) with grade 2 ILD at the time of treatment. Many patients with grade 3 ILD due to T-DXd treatment required steroid administration at the onset of ILD to improve prognosis. Additionally, the interval between discontinuation of T-DXd and initiation of the first post-T-DXd treatment was longer for patients with grade 3 ILD. Despite this, the proportion of cases in which ILD was not resolved at the start of the first post-T-DXd treatment was similar across all grades. However, these findings should be interpreted with caution because of the small number of patients who experienced grade 3 ILD.

The current Japanese T-DXd package insert indicates that patients who experience grade 1 ILD should discontinue T-DXd but may restart at a reduced dose level if all ILD findings have resolved and the therapeutic benefit is determined to greatly outweigh the risk [15]. However, at the time the EN-SEMBLE study was conducted, re-administration of T-DXd after ILD was not permitted. Therefore, only four patients underwent T-DXd rechallenge as the second post-T-DXd treatment.

Real-world data also suggest that the incidence of ILD in Japanese patients is higher than that in other countries [24,25]. An all-patient post-marketing surveillance (PMS) conducted in Japan with an observation period of 18 months continuously collected data following the launch of T-DXd to investigate the incidence of ILD. Among 1731 patients, the incidences of adjudicated drug-related ILD/pneumonitis of any grade, grade ≥3, and grade 5 were 16.1% (n = 278), 3.0% (n = 52), and 1.0% (n = 17), respectively [26]. The present analysis includes the safety and effectiveness results related to subsequent treatments in 146 of the cases included in the PMS. Thus, this analysis provides important insights because it includes follow-up data for over 50% of the patients with ILD who were included in the real-world PMS.

In patients with ILD caused by T-DXd, ILD grade affects the timing of the initiation of subsequent treatment, as well as treatment outcomes, but there was no difference in the choice of subsequent anti-HER2 therapy between grades. Post-T-DXd therapy was generally safe even after ILD. Recently, results of first-line T-DXd plus pertuzumab for HER2-positive mBC were reported, with adjudicated drug-related ILD occurring in 12.1% of patients [27]. Managing subsequent treatment in patients who develop T-DXd-related ILD raises important clinical questions when T-DXd is used as an earlier-line therapy. Therefore, the data from this subgroup analysis are expected to serve as valuable reference information for physicians when making treatment decisions in this context.

A strength of this analysis is that it was a large-scale study that comprehensively evaluated the effectiveness and safety of first post-T-DXd treatment in patients with mBC who developed ILD during T-DXd treatment in the real-world setting. A key limitation is the potential for selection bias, as the analysis included only cases of ILD caused by T-DXd in patients who were able to start subsequent therapies. Regarding the interpretation of treatment outcomes by ILD grade, it is also necessary to consider the breakdown of ILD types, such as diffuse alveolar damage, which has a poor prognosis, and organizing pneumonia, which has a better prognosis [28]. However, the EN-SEMBLE study did not take this aspect into account. The sample size of patients with grade 3 ILD was small, limiting the generalizability of the findings. In addition, because the EN-SEMBLE study was designed to evaluate outcomes of treatments administered after T-DXd, detailed information about the management and recovery course of ILD during T-DXd therapy (including steroid treatment for ILD and the subsequent clinical course) was not collected.

In conclusion, the present findings emphasize the importance of early detection and management of ILD in patients receiving T-DXd treatment for mBC to enable the achievement of optimal benefit with subsequent anti-HER2-targeted therapies. Importantly, the recurrence or exacerbation rate of ILD during subsequent lines of therapy was low among patients who had experienced ILD during T-DXd treatment.

## CRediT authorship contribution statement

Junji Tsurutani had full access to all study data and takes responsibility for the integrity of the data and the accuracy of the data analysis. **Junji Tsurutani:** Conceptualization, Methodology, Writing – original draft, Visualization, Investigation, Writing – review and editing, Formal analysis, Funding acquisition, Project administration, Resources, Supervision. **Kazuki Nozawa:** Conceptualization, Methodology, Writing – original draft, Visualization, Investigation, Writing – review and editing, Formal analysis. **Toru Mukohara:** Investigation, Writing – review and editing. **Tetsuhiko Taira:** Investigation, Writing – review and editing. **Akiyo Yoshimura:** Investigation, Writing – review and editing. **Shigenori E. Nagai:** Investigation, Writing – review and editing. **Jun Hashimoto:** Investigation, Writing – review and editing. **Kazuo Matsuura:** Investigation, Writing – review and editing. **Toshiro Mizuno:** Investigation, Writing – review and editing. **Yoshiaki Shinden:** Investigation, Writing – review and editing. **Mitsugu Yamamoto:** Investigation, Writing – review and editing. **Toshimi Takano:** Investigation, Writing – review and editing. **Makoto Wakahara:** Investigation, Writing – review and editing. **Hirofumi Terakawa:** Investigation, Writing – review and editing. **Takashi Yamanaka:** Investigation, Writing – review and editing. **Yasuyuki Kojima:** Investigation, Writing – review and editing. **Takahiro Nakayama:** Investigation, Writing – review and editing. **Yuji Hirakawa:** Conceptualization, Methodology, Writing – original draft, Visualization, Investigation, Writing – review and editing. **Kazuhito Shiosakai:** Investigation, Writing – review and editing, Data curation, Software, Validation, Formal analysis. **Hiroji Iwata:** Conceptualization, Methodology, Writing – original draft, Visualization, Investigation, Writing – review and editing, Formal analysis, Supervision.

## Prior publication

Data from this subanalysis have been presented at ESMO 2025 (Berlin, Germany, 17–21 October 2025).

## Data sharing statement

De-identified and anonymized study participant data underlying the results presented in this manuscript may be made available to researchers upon submission of a reasonable request to the corresponding author. The decision to share the de-identified/anonymized study data will be made by the corresponding author and the funder, Daiichi Sankyo Co., Ltd. Formal data sharing requests can be made up to 36 months from the article publication.

## Funding

This study was sponsored by Daiichi Sankyo Co., Ltd. The funding provider was involved in the study design, planning of the data analysis, data interpretation, and development of the manuscript but was not involved in data management and statistical analysis. In March 2019, AstraZeneca entered into a global development and commercialization collaboration agreement with Daiichi Sankyo for trastuzumab deruxtecan (T-DXd; DS-8201).

## Declaration of competing interests

All authors received support from Daiichi Sankyo Co., Ltd. for medical writing and the article processing charge for the submitted article. **Junji Tsurutani** received research funding from Daiichi Sankyo Co., Ltd., related to the submitted work; honoraria from Daiichi Sankyo Co., Ltd., AstraZeneca K.K., Eli Lilly Japan K.K., Pfizer Japan Inc., Eisai Co., Ltd., Kyowa Kirin Co., Ltd., Taiho Pharmaceutical Co., Ltd., MSD K.K., and Gilead Sciences, Inc.; and consulting fees from Daiichi Sankyo Co., Ltd., AstraZeneca K.K., Seagen Inc., Pfizer Japan Inc., and Gilead Sciences, Inc.; his institution received research funding from Eli Lilly Japan K.K., Kyowa Kirin Co., Ltd., Taiho Pharmaceutical Co., Ltd., Chugai Pharmaceutical Co., Ltd., MSD K.K., and Eisai Co., Ltd., outside the submitted work. **Kazuki Nozawa** received lecture fees from Daiichi Sankyo Co., Ltd., related to the submitted work; and lecture fees from Eli Lilly Japan K.K., Daiichi Sankyo Co., Ltd., and Chugai Pharmaceutical Co., Ltd., outside the submitted work. **Toru Mukohara** received research funding from Sysmex Corporation, Sanofi K.K., MSD K.K., Pfizer Japan Inc., Novartis Pharma K.K., Chugai Pharmaceutical Co., Ltd., AstraZeneca K.K., Ono Pharmaceutical Co., Ltd., Daiichi Sankyo Co., Ltd., and Gilead Sciences, Inc.; and lecture fees from Eisai Co., Ltd., Pfizer Japan Inc., Novartis Pharma K.K., Chugai Pharmaceutical Co., Ltd., Eli Lilly Japan K.K., AstraZeneca K.K., Kyowa Kirin Co., Ltd., Taiho Pharmaceutical Co., Ltd., and Daiichi Sankyo Co., Ltd., outside the submitted work. **Tetsuhiko Taira** received honoraria from Kyowa Kirin Co., Ltd., Chugai Pharmaceutical Co., Ltd., Eli Lilly Japan K.K., Pfizer Japan Inc., Taiho Pharmaceutical Co., Ltd., Daiichi Sankyo Co., Ltd., and MSD K.K., outside the submitted work. **Akiyo Yoshimura** received honoraria from Chugai Pharmaceutical Co., Ltd., AstraZeneca K.K., and Pfizer Japan Inc., outside the submitted work. **Shigenori E. Nagai** received research funding from Daiichi Sankyo Co., Ltd., related to the submitted work; honoraria from Eli Lilly Japan K.K., Pfizer Japan Inc., Daiichi Sankyo Co., Ltd., Chugai Pharmaceutical Co., Ltd., Eisai Co., Ltd., MSD K.K., Gilead Sciences, Inc., and Kyowa Kirin Co., Ltd.; and received consulting fees as a member of advisory boards funded by Daiichi Sankyo Co., Ltd. and Chugai Pharmaceutical Co., Ltd., outside the submitted work. **Jun Hashimoto** received honoraria from Daiichi Sankyo Co., Ltd., related to the submitted work, and Pfizer Japan Inc., AstraZeneca K.K., Eisai Co., Ltd., and Nippon Shinyaku Co., Ltd., outside the submitted work. **Kazuo Matsuura** received research funding from Eisai Co., Ltd. and lecture fees from Eisai Co., Ltd., Chugai Pharmaceutical Co., Ltd., Pfizer Japan Inc., Daiichi Sankyo Co., Ltd., Kyowa Kirin Co., Ltd., and AstraZeneca K.K., outside the submitted work. **Toshiro Mizuno** received research funding from Daiichi Sankyo Co., Ltd., related to the submitted work; and research funding from AstraZeneca K.K., Gilead Sciences, Inc., and Novartis Pharma K.K.; honoraria from AstraZeneca K.K., Pfizer Japan Inc., Ono Pharmaceutical Co., Ltd., Chugai Pharmaceutical Co., Ltd., Eli Lilly Japan K.K., MSD K.K., Eisai Co., Ltd., Novartis Pharma K.K., and Kyowa Kirin Co., Ltd.; and is a member of the Data Monitoring Committee funded by Taiho Pharmaceutical Co., Ltd., outside the submitted work. **Yoshiaki Shinden** received research funding from Daiichi Sankyo Co., Ltd., related to the submitted work; and lecture fees from Daiichi Sankyo Co., Ltd., Chugai Pharmaceutical Co., Ltd., Pfizer Japan Inc., Eisai Co., Ltd., Eli Lilly Japan K.K., Takeda Pharmaceutical Co., Ltd., Novartis Pharma Co., Ltd., Nippon Kayaku Co., Ltd., AstraZeneca K.K., and Kyowa Kirin Co., Ltd., outside the submitted work. **Mitsugu Yamamoto** received lecture fees from Pfizer Japan Inc., AstraZeneca K.K., Chugai Pharmaceutical Co., Ltd., and Merck & Co., Inc., outside the submitted work. **Toshimi Takano** received honoraria from Daiichi Sankyo Co., Ltd., Chugai Pharmaceutical Co., Ltd., and Eli Lilly Japan K.K., outside the submitted work. **Makoto Wakahara** received research funding from Daiichi Sankyo Co., Ltd., related to the submitted work; and lecture fees from Daiichi Sankyo Co., Ltd., Eli Lilly Japan K.K., Eisai Co., Ltd., AstraZeneca K.K., Pfizer Japan Inc., Kyowa Kirin Co., Ltd., MSD K.K., Gilead Sciences, Inc., Chugai Pharmaceutical Co., Ltd., and Taiho Pharmaceutical Co., Ltd., outside the submitted work. **Hirofumi Terakawa** received research funding from Chugai Pharmaceutical Co., Ltd. and honoraria from Daiichi Sankyo Co., Ltd., Chugai Pharmaceutical Co., Ltd., MSD K.K., Eli Lilly Japan K.K., Taiho Pharmaceutical Co., Ltd., Kyowa Kirin Co., Ltd., and Eisai Co., Ltd., outside the submitted work. **Takashi Yamanaka** received consulting fees from Daiichi Sankyo Co., Ltd. and Eli Lilly Japan K.K.; honoraria from AstraZeneca K.K., Chugai Pharmaceutical Co., Ltd., Eli Lilly Japan K.K., Daiichi Sankyo Co., Ltd., Kyowa Kirin Co., Ltd., Pfizer Japan Inc., Taiho Pharmaceutical Co., Ltd., and Gilead Sciences, Inc., outside the submitted work. **Yasuyuki Kojima** received honoraria from Daiichi Sankyo Co., Ltd., AstraZeneca K.K., Kyowa Kirin Co., Ltd., Chugai Pharmaceutical Co., Ltd., Biomaster, Inc., Exact Sciences Corp., MSD K.K., and Taiho Pharmaceutical Co., Ltd. and lecture fees from Daiichi Sankyo Co., Ltd. and Eisai Co., Ltd., outside the submitted work. **Takahiro Nakayama** received lecture fees from Chugai Pharmaceutical Co., Ltd., Eli Lilly Japan K.K., Novartis Pharma K.K., AstraZeneca K.K., Pfizer Japan Inc., Taiho Pharmaceutical Co., Ltd., Daiichi Sankyo Co., Ltd., Eisai Co., Ltd., MSD K.K., Sandoz K.K., Celltrion Healthcare Japan K.K., Yakult Honsha Co., Ltd., and Nippon Kayaku Co., Ltd., outside the submitted work. **Yuji Hirakawa** and **Kazuhito Shiosakai** are employees of Daiichi Sankyo Co., Ltd. **Hiroji Iwata** received consulting fees and honoraria from Daiichi Sankyo Co., Ltd., Chugai Pharmaceutical Co., Ltd., AstraZeneca K.K., Eli Lilly Japan K.K., MSD K.K., and Pfizer Japan Inc.; consulting fees from Gilead Sciences, Inc.; and honoraria from Taiho Pharmaceutical Co., Ltd. and Kyowa Kirin Co., Ltd.; and his institution received research funding from Chugai Pharmaceutical Co., Ltd., Daiichi Sankyo Co., Ltd., and AstraZeneca K.K., outside the submitted work.

## Glossary

CDK4/6i: CDK4/6 inhibitors
CI: confidence interval
HER2: human epidermal growth factor receptor 2
ILD: interstitial lung disease
mBC: metastatic breast cancer
NE: not estimable
OS: overall survival
PD: progressive disease
PMS: post-marketing surveillance
rwPFS: real-world progression-free survival
rwTTF: real-world time to treatment failure
rwTTNT: real-world time to next treatment
T-DXd: trastuzumab deruxtecan
