# Supplementary material for: Safety and effectiveness of post-trastuzumab deruxtecan regimens in patients with HER2-positive metastatic breast cancer who discontinued trastuzumab deruxtecan due to interstitial lung disease
Source: Breast. 2026 Feb 4;86:104722. doi: 10.1016/j.breast.2026.104722 (PMC12966697; doi:10.1016/j.breast.2026.104722)
Supplement: Multimedia component 1 [file mmc1.docx]

**Supplementary Materials**

**Supplementary Table 1. Distribution of treatment regimens following T-DXd treatment**

|  | **ILD subgroup**  **(any grade)** | **ILD grade for discontinuation of T-DXd treatment^a,b^** | | |  |
| --- | --- | --- | --- | --- | --- |
|  |  | **Grade 1** | **Grade 2** | **Grade ≥3** | |
| No. patients, n (%) | 146 (100) | 78 (53.4) | 46 (31.5) | 18 (12.3) | |
| Anti-HER2 therapy^c^ | 119 (81.5) | 63 (80.8) | 36 (78.3) | 16 (88.9) | |
| Anti-HER2 antibody | 96 (65.8) | 49 (62.8) | 31 (67.4) | 12 (66.7) | |
| Trastuzumab with pertuzumab | 54 (37.0) | 25 (32.1) | 15 (32.6) | 11 (61.1) | |
| Trastuzumab without pertuzumab | 42 (28.8) | 24 (30.8) | 16 (34.8) | 1 (5.6) | |
| HER2-TKI | 20 (13.7) | 12 (15.4) | 4 (8.7) | 4 (22.2) | |
| Lapatinib | 20 (13.7) | 12 (15.4) | 4 (8.7) | 4 (22.2) | |
| ADC | 3 (2.1) | 2 (2.6) | 1 (2.2) | 0 | |
| Trastuzumab emtansine | 3 (2.1) | 2 (2.6) | 1 (2.2) | 0 | |
| Non-anti-HER2 therapy | 27 (18.5) | 15 (19.2) | 10 (21.7) | 2 (11.1) | |
| Chemotherapy | 10 (6.8) | 6 (7.7) | 4 (8.7) | 0 | |
| Chemotherapy + bevacizumab | 0 | 0 | 0 | 0 | |
| Endocrine therapy (monotherapy or with a CDK4/6i) | 16 (11.0) | 9 (11.5) | 5 (10.9) | 2 (11.1) | |
| Other | 1 (0.7) | 0 | 1 (2.2) | 0 | |

Data are shown as n (%).

ADC, antibody–drug conjugate; CDK4/6/i, cyclin-dependent kinase 4/6 inhibitor; HER2, human epidermal growth factor receptor 2; ILD, interstitial lung disease; T-DXd, trastuzumab deruxtecan; TKI, tyrosine kinase inhibitor

^a^Grade determined by the attending physician.

^b^There were no patients with known grade 4 or 5 ILD; the ILD grade of four patients was unknown.

^c^Combination treatment regimens including anti-HER2 therapy alongside chemotherapy, endocrine therapy, or CDK4/6i were classified as anti-HER2 therapy.
